# Validation of *COL11A1*/procollagen 11A1 expression in TGF-β1-activated immortalised human mesenchymal cells and in stromal cells of human colon adenocarcinoma

**DOI:** 10.1186/1471-2407-14-867

**Published:** 2014-11-23

**Authors:** José A Galván, Jorge García-Martínez, Fernando Vázquez-Villa, Marcos García-Ocaña, Carmen García-Pravia, Primitiva Menéndez-Rodríguez, Carmen González-del Rey, Luis Barneo-Serra, Juan R de los Toyos

**Affiliations:** Surgery Department, School of Medicine and Health Sciences, University of Oviedo, 33006 Oviedo, Spain; Oncology University Institute of the Principality of Asturias (IUOPA), 33006 Oviedo, Spain; Preparative Biotechnology Unit, Technical-Scientific Services, University of Oviedo, 33006 Oviedo, Spain; Pathological Anatomy Service, Asturias Central University Hospital (HUCA), 33006 Oviedo, Spain; Immunology Department, School of Medicine and Health Sciences, University of Oviedo, c/ Julián Clavería s/n, 33006 Oviedo, Spain; Translational Research Unit (TRU), Institute of Pathology, University of Bern, Bern, Switzerland

**Keywords:** Procollagen 11A1, Human bone marrow mesenchymal cells, Cancer-associated stromal cells, Human colon adenocarcinoma

## Abstract

**Background:**

The human *COL11A1* gene has been shown to be up-regulated in stromal cells of colorectal tumours, but, so far, the immunodetection of procollagen 11A1, the primary protein product of *COL11A1*, has not been studied in detail in human colon adenocarcinomas. Some cancer-associated stromal cells seem to be derived from bone marrow mesenchymal cells; the expression of the *COL11A1* gene and the parallel immunodetection of procollagen 11A1 have not been evaluated in these latter cells, either.

**Methods:**

We used quantitative RT-PCR and/or immunocytochemistry to study the expression of *DES/*desmin, *VIM/*vimentin, *ACTA2/*αSMA (alpha smooth muscle actin) and *COL11A1*/procollagen 11A1 in HCT 116 human colorectal adenocarcinoma cells, in immortalised human bone marrow mesenchymal cells and in human colon adenocarcinoma-derived cultured stromal cells. The immunodetection of procollagen 11A1 was performed with the new recently described DMTX1/1E8.33 mouse monoclonal antibody. Human colon adenocarcinomas and non-malignant colon tissues were evaluated by immunohistochemistry as well. Statistical associations were sought between anti-procollagen 11A1 immunoscoring and patient clinicopathological features.

**Results:**

Procollagen 11A1 was immunodetected in human bone marrow mesenchymal cells and in human colon adenocarcinoma-associated spindle-shaped stromal cells but not in colon epithelial or stromal cells of the normal colon. This immunodetection paralleled, in both kinds of cells, that of the other mesenchymal-related biomarkers studied: vimentin and alpha smooth muscle actin, but not desmin. Thus, procollagen 11A1^+^ adenocarcinoma-associated stromal cells are similar to “activated myofibroblasts”. In the series of human colon adenocarcinomas here studied, a high procollagen 11A1 expression was associated with nodal involvement (p = 0.05), the development of distant metastases (p = 0.017), and advanced Dukes stages (p = 0.047).

**Conclusion:**

The immunodetection of procollagen 11A1 in cancer-associated stromal cells could be a useful biomarker for human colon adenocarcinoma characterisation.

**Electronic supplementary material:**

The online version of this article (doi:10.1186/1471-2407-14-867) contains supplementary material, which is available to authorized users.

## Background

A wealth of studies have reported that the *COL11A1* human gene, coding for the α1 chain of procollagen and mature collagen of type XI, which is an extracellular minor fibrillar collagen, is up-regulated in some human tumours and in mesenchymal-derived tumour cell lines [1–32], as well as in mesenchymal stem cells and osteoblasts [33–35].

Collagen polypeptides are synthesized as procollagens, with the N- and C-propeptides at the ends of the prototypical collagen triple helix. Upon secretion, the propeptides are excised and then the mature collagen molecules assemble in fibrils.

In tumours, the expression of the *COL11A1* gene is currently associated to a fibroblast-like stromal phenotype [12, 19] but the origin and nature of the cells which produce procollagen and collagen 11A1 remain controversial to some extent [26].

The so-called cancer-associated stromal cells, resulting from the desmoplastic reaction which accompanies the development of human invasive carcinomas, comprise cells of different types, and are at least in part derived from mesenchymal progenitors and local resident cells. It is also well-established that TGF-β1 in cancer promotes the activation of cancer-associated stromal cells [36].

For the present study, we set out to verify the expression of the *COL11A1* gene, by quantitative RT-PCR in TGF-β1-exposed epithelial human colorectal HCT 116 cells and Immortalised Human Bone Marrow Mesenchymal Cells (hTERT-HMCs); and the expression of procollagen 11A1 by immunocytochemistry (ICC)/immunohistochemistry (IHC), using the DMTX1/1E8.33 monoclonal antibody (mAb) [37], on those cell cultures as well as on biopsies of human colon adenocarcinomas. Concurrently, we studied the expression of *DES/*desmin, *VIM/*vimentin and *ACTA2/*αSMA (alpha smooth muscle actin) as mesenchymal (myofibroblast)/stromal markers.

Within the N-propeptide of human procollagen 11A1, it is the so-called “variable region”, the most divergent amino acid sequence stretch among different procollagens. The DMTX1/1E8.33 mAb recognises an epitope in the YNYGTMESYQTEAPR amino acid stretch within the variable region of human procollagen 11A1 [37].

## Methods

### Cell cultures

Ascorbate is a well-known inducer of the synthesis of some collagens [38, 39]; thus, to favour the expression of procollagen 11A1, cells were habitually cultured with this supplement. Since TGF-β1 levels are increased in the serum of patients with invasive carcinomas [40], we chose to analyse its effects after continued and protracted exposure of cell cultures to this cytokine.

The human colorectal adenocarcinoma HCT 116 (CCL-247) cell line, derived from a primary tumour, was obtained from the American Type Culture Collection (ATCC) and cultured in DMEM, supplemented with 1 mM sodium pyruvate (Biochrom), 2 mM L-glutamine (Biochrom), 1X non-essential amino acids (Biochrom), 10% foetal bovine serum (Biochrom), and ascorbate 2-phosphate (37.5 μg/ml) (Wako Chemicals).

Immortalised Human Bone Marrow Mesenchymal Cells-hTERT (hTERT-HMCs) were obtained from Applied Biological Materials (ABM) Inc., Richmond, BC, Canada (Cat. No. T0523), and grown in T25 ECM-coated flasks in Prigrow II medium (ABM, Cat. No. TM002), with the addition of 10% foetal bovine serum, 1 μM hydrocortisone (Sigma) and ascorbate 2-phosphate (37.5 μg/ml) (Wako Chemicals).

For TGF-β1 induction, media were further supplemented with 10 ng/ml of recombinant TGF-β1 (Peprotech). The medium was replaced every 3–4 days and the cells were cultured for at least 15 days.

All the cultures were carried out in a humidified atmosphere of 5% CO_2_ in air at 37°C.

Culture passages and cell collections were done with trypsin/EDTA 0.05%/0.02% (Biochrom). Three different harvests from each cell culture type were obtained; for Q-RT-PCR, fresh cell pellets were kept at -80°C.

### Colon adenocarcinoma stromal cells isolation and culture

Fresh human tissue samples were procured after written informed consent of the patients and approval by the Principality of Asturias Ethics Committee of Clinical Research, Oviedo, Spain.

Short-term cultures of colon adenocarcinoma stromal cells were carried out, as previously described [41], from samples of tumoral sites, avoiding necrotic areas. A sample from the operating theatre was directly transferred to a sterile tube containing DMEM culture medium (Gibco, Invitrogen), supplemented with vancomycin (40 μg/ml) and amikacin (40 μg/ml) (Normon Laboratories, Madrid, Spain), and stored for 24 hours at 4°C.

After three washings with phosphate buffer saline (PBS), the sample was cut into several small fragments. These fragments were first incubated with collagenases (Type I 2 mg/ml, Sigma) for 1.5 hours and then centrifuged to eliminate supernatant; subsequently, the pellet was subjected to a second incubation in trypsin/EDTA for 30 min. After digestion, the cells were again collected in a pellet, resuspended in DMEM culture medium, supplemented with 10% foetal bovine serum, L-glutamine and penicillin/streptomycin, transferred to T-flasks and cultivated in 5% CO_2_ at 37°C.

Stromal cell cultures were stable up to 5–6 passages before going into senescence. The purity of these stromal cell cultures was assessed by morphology and by immunostaining for vimentin.

### Q-RT-PCR

For normalisation of data, quantitative RT-PCR of *DES, VIM, ACTA2 and COL11A1* mRNA, and *PUM1*, *RPL10*, and *GAPDH* mRNA was performed using the BioMark™ HD System of the Fluidigm technology (Fluidigm, San Francisco, USA).

Briefly, total RNA was isolated from pooled cell cultures, kept at -80°C, with the RNeasy Mini kit (Qiagen). cDNA was synthesized from 100 ng of RNA from each sample, using the AffinityScript Multiple Temperature cDNA Synthesis kit (Agilent Technologies). A pre-amplification was carried out, applying the QIAGEN® Multiplex PCR Kit and the pool of all the 20x TaqMan® Gene Expression Assays. Real time Q-PCR reactions were carried out with the TaqMan Universal PCR Master Mix kit (Applied Biosystems). Further details, according to Applied Biosystems’ recommendations, are in Table 1.

**Table 1:** **Assays selected and PCR conditions for Q-RT-PCR of mRNA analysis**

Gene	Assay ID
COL11A1	Hs01097664_m1
GAPDH	Hs02758991_g1
PUM1	Hs004472881_m1
RPL10	Hs00749196_s1
DES	Hs00157258_m1
ACTA2	Hs00426835_g1
VIM	Hs00185584_m1

PCR conditions were: 50°C – 2 min; 95°C – 10 min; and 40 amplification cycles:

95°C – 15 sec and 60°C – 1 min.

Data were normalised by applying the ΔCt method, after PCR efficiency corrections. These analyses were performed by Progenika Biopharma, S.A., Derio, Spain.

Three independent samples (*n* =3) of different cell harvests of each cell type were studied. Data are presented as mean and SEM. For each gene, differences between cell culture expressions were analysed by a two-tailed unpaired *t-*test. A *P* value <0.01 was considered statistically significant.

### Immunohistochemistry (IHC)

For immunohistochemical techniques, a cohort of 51 patients with colon adenocarcinoma and 6 patients diagnosed with incipient bowel infarction were collected from the Archive of the Pathology Department, Asturias Central University Hospital, with the Principality of Asturias Ethics Committee of Clinical Research, Oviedo, Spain, approval for guidelines on ethical procedures. The samples had been fixed with 10% formaldehyde for 24 h and embedded in paraffin.

Three-μm thick tissue sections were stained with Hematoxylin and Eosin (H&E) for histological examination. Antigen retrieval was performed by heating in *PTLink* (DakoCytomation, Denmark) in buffer solution at high pH for 20 minutes. Endogenous peroxidase activity was blocked with *Peroxidase Blocking Reagent* (DakoCytomation, Denmark) for 5 minutes. After that, samples were first incubated at 37°C with the primary antibodies described in Table 2. Subsequently, the EnVision system (HRP Flex) (DakoCytomation) was applied for 30 minutes at room temperature. Then, the samples were stained with DAB (3-3′-Diaminobenzidine) (DakoCytomation, Denmark) for 10 minutes, counterstained for 10 minutes with hematoxylin (DakoCytomation), dehydrated and mounted in Entellan® (Merck, Germany). Finally, the stained tissue sections were studied and photographed (40× objective) under a light microscope (Nikon - Eclipse 80i).

**Table 2:** **Antibodies used in IHC/ICC analysis**

Primary antibodies (species)	Clone	Commercial reference	Dilution	Incubation time (min)
Procollagen 11A1 (mAb)	1E8.33	DMTX1/Oncomatrix, Spain	1:400	30
Desmin (mAb)	D33	Dako, Denmark	R-t-U	20
α-SMA (mAb)	1A4	Dako, Denmark	R-t-U	20
Vimentin (pAb)	C-20	Santa Cruz Biotech, Germany	1:600	10

*mAb*: Mouse monoclonal antibody.

*pAb*: Rabbit polyclonal antibodies.

*R-t-U*: Ready-to-Use.

### Immunocytochemistry (ICC)

Cells were fixed in 10% formaldehyde for 10 minutes in the chamber slide (BD Falcon™, ref. 354114). Endogenous peroxidase activity was blocked with *Peroxidase Blocking Reagent* (DakoCytomation, Denmark) for 5 minutes. Permeabilization step was performed adding wash buffer 1× (DakoCytomation, Denmark) which contains 0.05 mol/L Tris/HCl, 0.15 mol/L NaCl, 0.05% Tween-20 [41]. Primary antibodies were applied, as described in Table 2, at room temperature. After that, slides were incubated with the EnVision system (HRP Flex) for 10 minutes at room temperature. Then, the samples were visualised with DAB for 5 minutes, and counterstained with hematoxylin for 5 minutes. Finally, the stained slides were dehydrated, mounted, studied and photographed as above.

### Immunohistochemistry assessment

Specimens were assessed by three observers (JAG, CGP and CGR), following these criteria: procollagen 11A1 immunostaining was evaluated according to the cytoplasmatic signal as the product of two parameters: *extent of immunoreactivity*, which was evaluated in the most densely stained area (hot spot) under the 20× objective and scored on a 0–3 scale, according to the proportion of positive fibroblasts: (0) 0%; (1) <10%; (2) 10-50% and (3) >50%; and *granularity in the cytoplasm*, evaluated as dispersed vs. confluent (1 and 2 points, respectively), with the 40× objective. Immunoscore values ranged from 0 to 6. Adjacent non-malignant tissue was used as a negative control.

### Statistical analysis

The experimental results were tested for significance employing the χ^2^ test (with Yates’ correction, when appropriate). The statistical analysis was carried out with the IBM SPSS 20.0 software package (SPSS, Inc., Chicago, IL). All tests were two-sided and *p* < 0.05 values were considered statistically significant.

## Results

### Study of human cell cultures

So far, we are not aware of any human colorectal cell line in which the expression of the *COL11A1* gene has been reported, but we had observed that primary cultures of bone marrow-derived human mesenchymal cells, expressed *COL11A1*/procollagen 11A1, especially after long exposure (≥15 days) to TGF-β1 (data not shown).

We have presently studied the well-known epithelial human colorectal HCT 116 cell line as a negative control for the expression of the mesenchymal *DES*, *VIM*, *ACTA2* and *COL11A1* genes in relation to their expression by cultured immortalised hTERT-HMCs. The expression of the *DES*, *VIM*, *ACTA2* and *COL11A1* genes was analysed by Q-RT-PCR; the immunodetection of desmin, vimentin, αSMA and procollagen 11A1 was performed by ICC.

**Figure 1:**
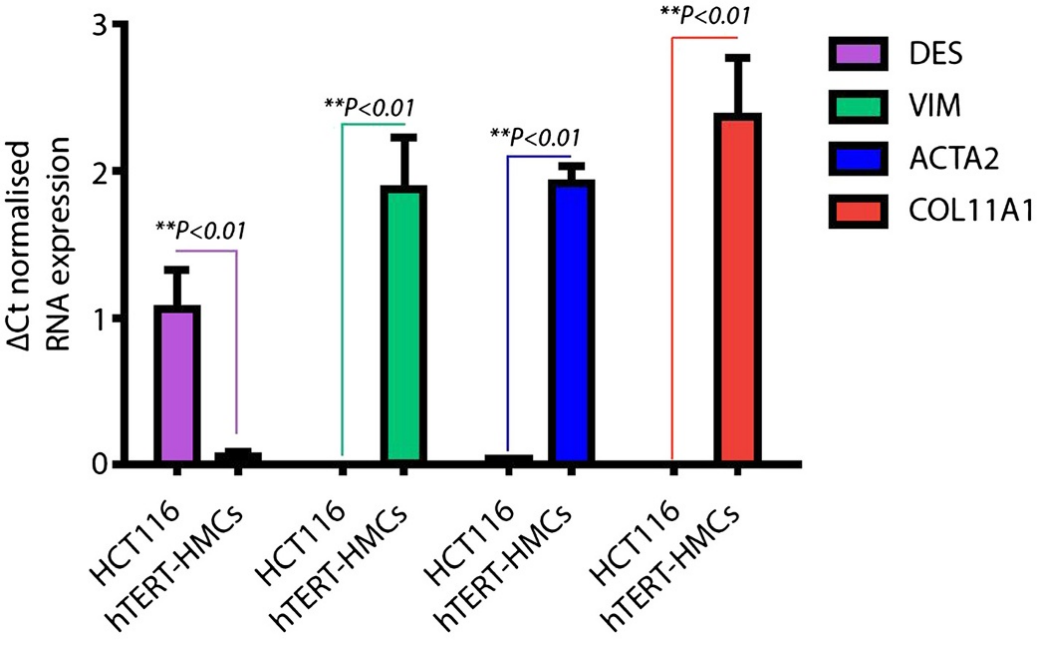
**Q-RT-PCR data of** ***DES*** **,** ***VIM*** **,** ***ACTA2 and COL11A1*** **mRNA expression in cell cultures of the HCT 116 cell line and in immortalised hTERT-HMCs, both after long exposure to ascorbate 2-phosphate and TGF-β1.** The data were normalised in relation to *PUM1*, *RPL10*, and *GAPDH* mRNA expression (n =3; mean ± SEM; *P <0.05, **P <0.01).

**Figure 2:**
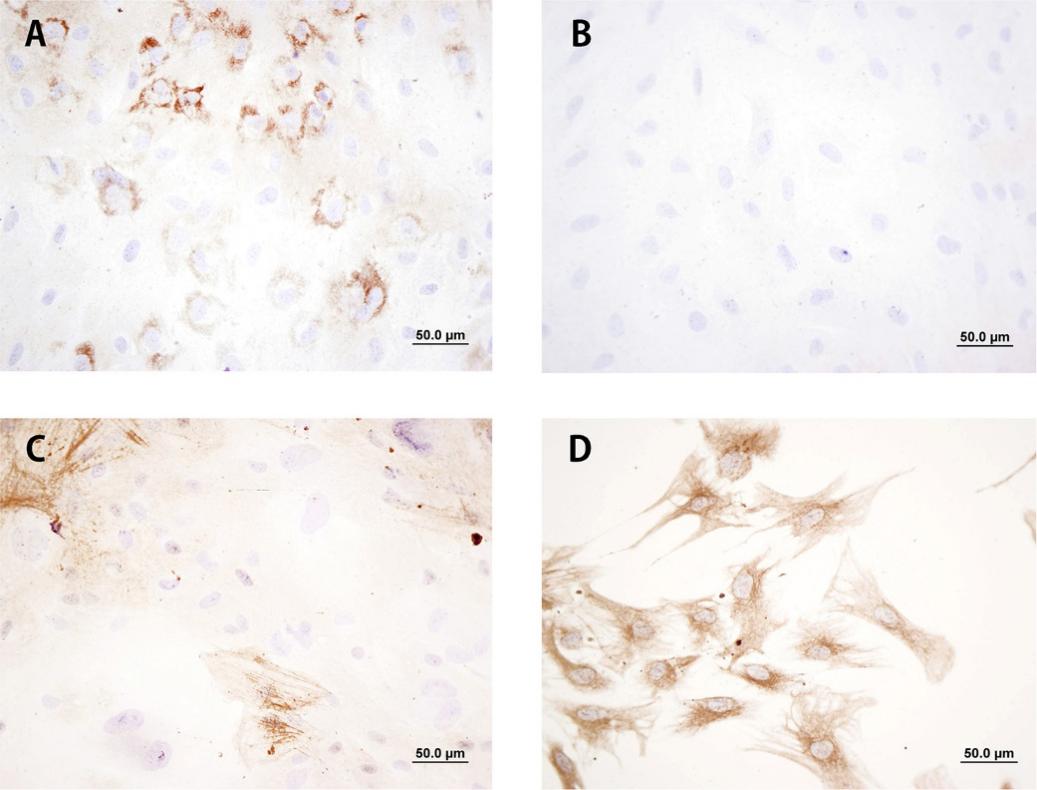
**Representative immunostaining of cultured immortalised hTERT-HMCs after long exposure to ascorbate 2-phosphate and TGF-β1. A)** Procollagen 11A1 **B)** Desmin, **C)** αSMA and **D)** Vimentin. Scale bar 50 μm (400×).

According to the normalised Q-RT-PCR data we have obtained (Figure 1), TGF-β1-activated hTERT-HMCs did not express *DES* mRNA, but noticeable amounts of *VIM, ACTA2 and COL11A1* mRNA. The corresponding protein expression was confirmed by ICC (Figure 2). An average of 20% of these cells in cultures exposed to TGF-β1 showed a granular pattern of intracytoplasmic immunostaining of procollagen 11A1. None of these markers was expressed by the HCT 116 cells, but certain levels of *DES*.

### Examination of human tissues

**Figure 3:**
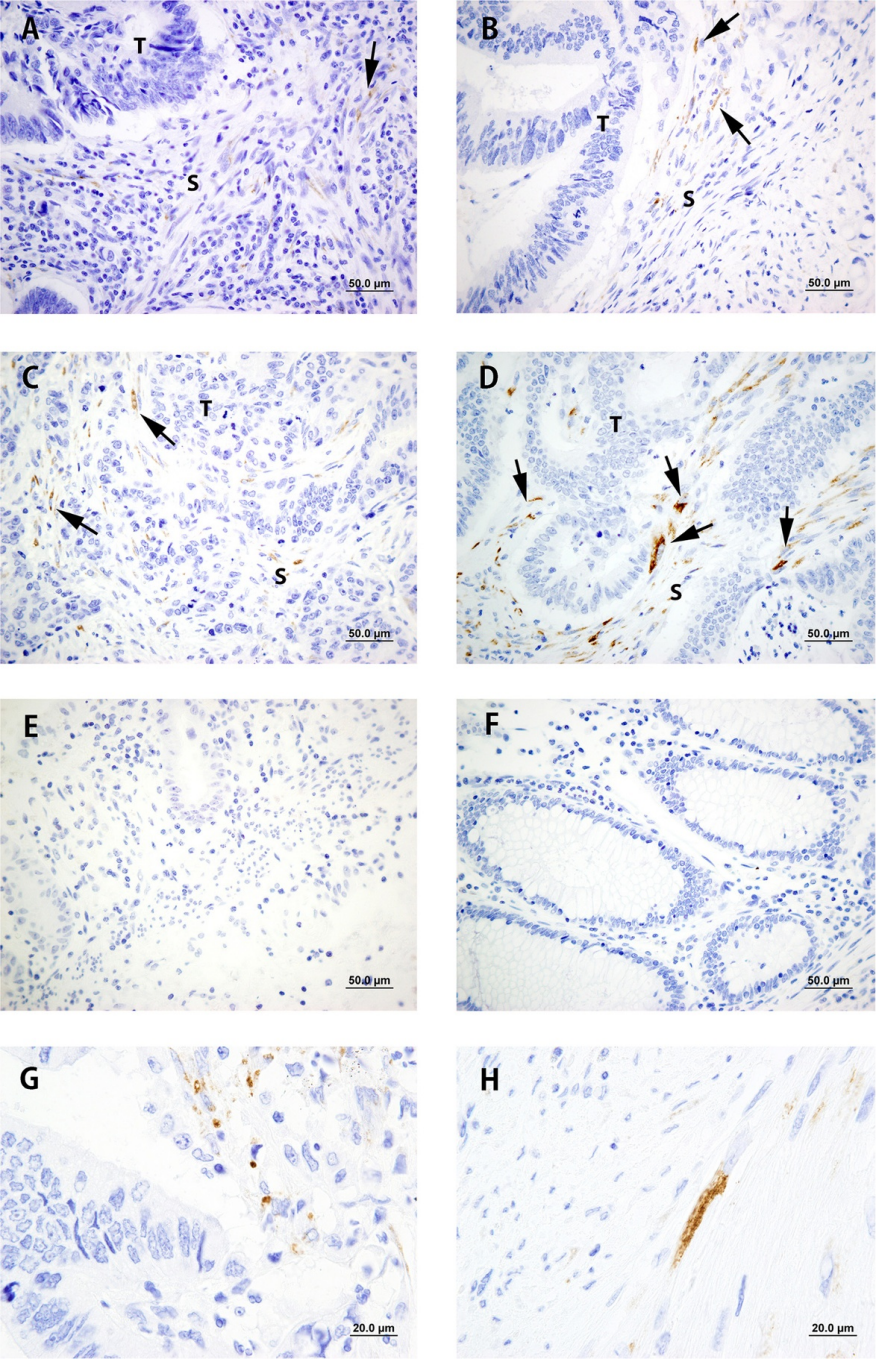
**Representative procollagen 11A1 immunostaining in colon adenocarcinoma. A)** Score 1, **B)** Score 2; **C)** Score 4; **D)** Score 6. Arrow heads point to stained peritumoral stromal cells. **E)** Bowel ischemia; **F)** Non-malignant tissue. Scale bar 50 μm (400X). **G)** Dispersed granularity and **H)** Confluent granularity. Scale bar 20 μm (1000×).

**Figure 4:**
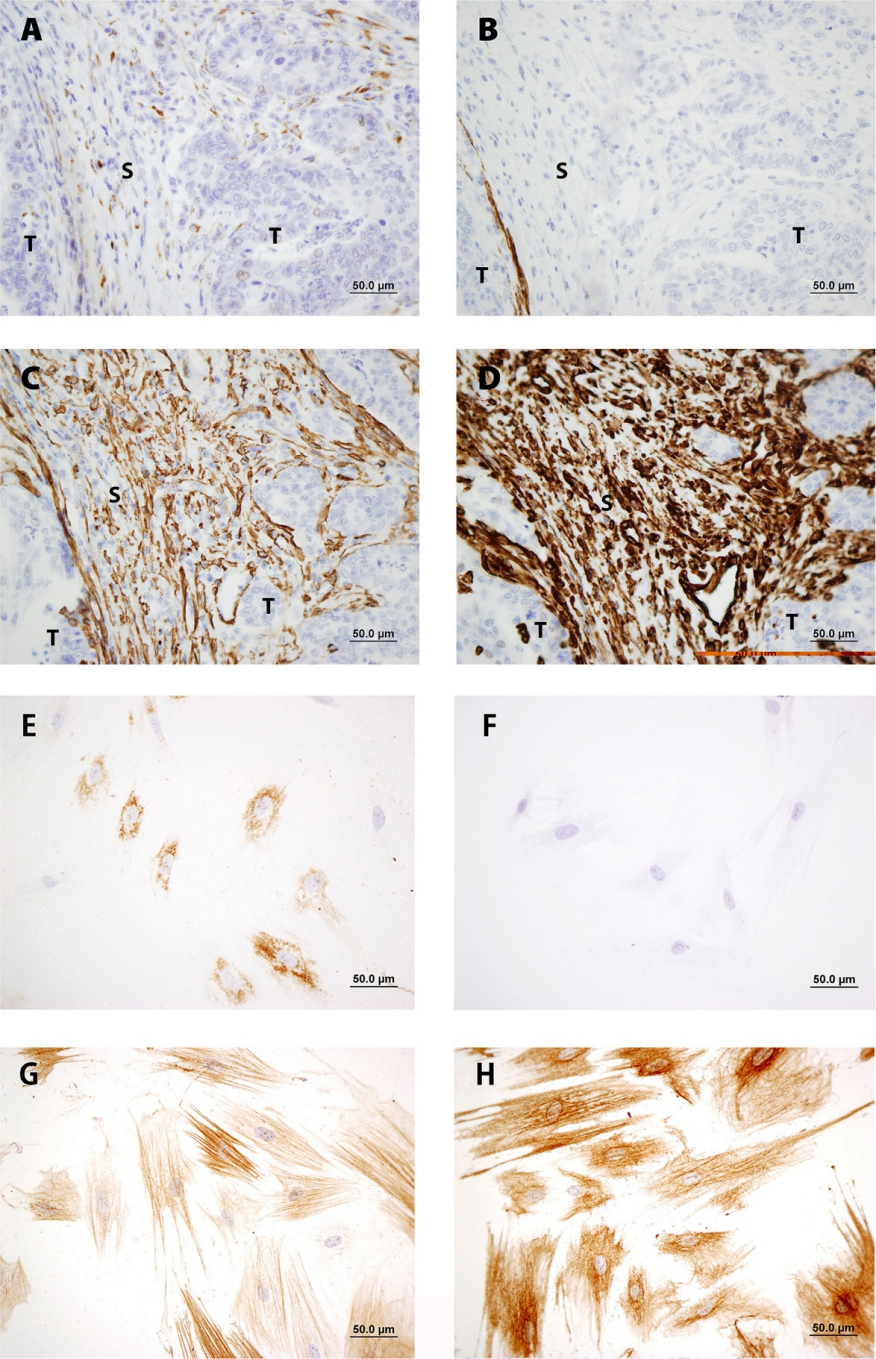
**Representative immunostaining of a colon adenocarcinoma: A) Procollagen 11A1 (immunoscore 6), B) Desmin, C) αSMA and D) Vimentin (these images were taken from the same area of serial sections; and of cultured stromal cells from the same case: E) Procollagen 11A1, F) Desmin, G) αSMA and H) Vimentin.** Scale bar 50 μm (400×).

Fifty one paraffin-embedded archival samples of human colon adenocarcinomas were examined by IHC with the DMTX1/1E8.33 mAb; all cases presented adjacent non-malignant tissue as control. Six cases of incipient bowel infarction were similarly studied. Table 3 shows the characteristics of patients and samples, and their anti-procollagen 11A1 immunoscores, evaluated as described in Methods; as shown, these immunoscores ranged from 0 to 6. A more detailed description of these characteristics is in Additional file 1. In three of these 51 diagnosed adenocarcinoma cases, no procollagen 11A1 staining could be detected, in spite of extensive re-examination. Procollagen 11A1 was neither immunodetected in the adjacent non-malignant tissues nor in the infarction cases.Figure 3 shows representative procollagen 11A1 immunostaining patterns (panels A, B, C and D); only a granular cytoplasmic staining of peritumoral spindle-shaped fibroblast-like stromal cells was observed. This granularity was either dispersed, with a few granules in the cytoplasm of stromal cells (panel G) or frankly confluent (panel H). No staining was observed on specimens of bowel ischemia (panel E) or on non-malignant tissues (panel F).Figure 4 shows a representative immunostaining of an adenocarcinoma specimen with a procollagen 11A1 immunoscore of 6. As shown on panel A, only peritumoral stromal cells were stained with the anti-procollagen 11A1 mAb; besides, these cells seemed to be positive for αSMA (C) and vimentin (D), but negative for desmin (B). This immunostaining pattern was reproduced (panels E, F, G and H) in stromal cells cultured from fresh specimens of the same patient.

**Table 3:** Patient characteristics (N = 51)*

		Frecuency N	(%)
**Gender**	Female	19	37.3
	Male	32	62.7
**Age (years)**	Median (range)	70	(31–85)
**Tumor size (cm)**	Median (range)	3.7	(0.5 - 11)
**Localization**	Ascending colon	21	41.2
	Descending colon	8	15.7
	Sigmoid	22	43.1
**Differentiation**	Well differentiated	19	37.3
	Moderately differentiated	28	54,9
	Poorly differentiated	4	7.8
**T**	T1	3	5,9
	T2	7	13.7
	T3	28	54.9
	T4	13	25.5
**N**	pN0	25	49.0
	pN1	26	51.0
**M**	M0	39	76.5
	M1	12	23.5
**TNM staging**	I	8	15,7
	IIA	12	23,5
	IIB	4	7,8
	IIIA	1	2,0
	IIIB	9	17,6
	IIIC	5	9,8
	IV	12	23,5
**Dukes staging**	A	8	15,7
	B	16	31,4
	C	15	29,4
	D	12	23,5
**Anti-procollagen 11A1 immunostaining by score**	0	3	5.9
	1	12	23.5
	2	13	25.5
	3	4	7.8
	4	6	11.8
	6	13	25.5
**Anti-procollagen 11A1 immunostaining**	Low (≤2)	28	54.9
**(Median =2)**	High (>2)	23	45.1

(*) Patients diagnosed with colon adenocarcinoma.

Patients diagnosed with ischemia were excluded**.**

### Association between procollagen 11A1 expression and clinicopathological features

For statistical purposes, some variables (age at diagnosis, tumour size, anti-procollagen 11A1 immunostaining) were divided into 2 groups, taking the median score value as a cut-off point (Table 4). Patients diagnosed with ischemia were excluded from this analysis.

**Table 4:** **Association between procollagen 11A1 expression and clinicopathological features**

	Anti-procollagen 11A1 immunostaining (Median =2)			
		Low (≤2)	High (>2)	***p***
**Age (Median 70 years)**	≤ 70 years	13	14	0.304
	> 70 years	15	9	
**Gender**	Female	18	14	0.802
	Male	10	9	
**Localization**	Ascending colon	9	12	0.260
	Descending colon	6	2	
	Sigmoid	13	9	0.200
**Tumor size (Median = 3.7 cm)**	Small ≤3.7 cm	12	14	
	Large >3.7 cm	16	9	0.370
**Differentiation**	Well differentiated	12	7	
	Moderately differentiated	15	13	
	Poorly differentiated	1	3	
**T**	T1-T2	8	2	0.075
	T3-T4	20	21	
**N**	Absent	17	8	
	Present	11	15	**0.059**
**M**	Absent	25	14	
	Present	3	9	**0.017**
**Stage grouping**	I	7	1	0.141
	IIA	7	5	
	IIB	2	2	
	IIIA	1	0	
	IIIB	6	3	
	IIIC	2	3	
	IV	3	9	
**Dukes staging**	A	7	1	
	B	9	7	
	C	9	6	**0.047**
	D	3	9	

9/12 patients that had developed distant metastases at diagnosis and 15/27 patients with advanced Dukes stages were associated with high procollagen 11A1 expression (*p* = 0.017 and *p* = 0.047, respectively). The same can be observed for 15/26 patients with nodes affected, however, only with a trend towards significance (*p* = 0.059).

## Discussion

We have presently shown, extending our previous observations [42], that procollagen 11A1, as a protein expression product of the *COL11A1* gene, is immunodetected in stromal cells of human colon adenocarcinoma. By contrast, and contrary to a previous report [15], we have never observed immunodetection of procollagen 11A1 in epithelial cells of normal colon tissue or colon adenocarcinoma with the DMTX1/1E8.33 mAb. This immunodetection was observed in 48 of the 51 cases studied. Three cases (5.9%), which were classified under the same criteria as the rest of the cases examined as conventional adenocarcinomas with desmoplastic reaction, did not stain; so far, we have not identified in them any characteristics to which this negative immunostaining could be associated. Except for the report of Fischer *et al*. [1] who did not find either the expression of *COL11A* in 5 out of 15 (33.3%) colonic carcinomas analysed, we are not aware of any more studies reporting the percentage of colon adenocarcinomas expressing the *COL11A1* gene; this aspect should be studied in detail.

We have very recently reported that procollagen 11A1^+^ cancer-associated stromal cells of pancreatic ductal adenocarcinoma co-express αSMA, and/or vimentin, and/or desmin in different proportions [41]. We have now confirmed, by Q-RT-PCR and IHC/ICC, the stromal expression of human procollagen 11A1 in colon adenocarcinoma. Although not formally proven, procollagen 11A1^+^ colon adenocarcinoma stromal cells, being spindle-shaped, seem to simultaneously express alpha smooth muscle actin and vimentin, but no desmin; these traits confer to them a myofibroblast-like phenotype rather than a pericyte one [43]. While in the desmoplastic component of hepatocellular carcinomas and pancreatic ductal adenocarcinomas there is a significant contribution of desmin + stellate cells, this is not the case in colon adenocarcinomas. As normal resident intestinal myofibroblasts are not immunoreactive to the DMTX1/1E8.33 mAb, these procollagen 11A1^+^ desmin^-^ colon adenocarcinoma stromal cells could be a type of “activated myofibroblasts”.

We have also shown that a fraction of cultured immortalised HMCs, after long exposure to TGF-β1, exhibit a very similar phenotype to the described above for cancer-associated stromal cells. It is intriguing that only as much as 20% of these cultured cells express procollagen 11A1; this aspect warrants further analysis as well as the global genotype and phenotype of procollagen 11A1^+^ cells.

It has been reported that human bone marrow-derived mesenchymal cells may differentiate *in vitro* to fibroblast/myofibroblast-like cells under certain conditions, such as coculture with human colon carcinoma cells and TGF-β1, or prolonged exposure to conditioned medium from MDA-MB-231 breast cancer cells; these fibroblast/myofibroblast-like cells are able to promote tumour growth both *in vitro* and *in vivo* [36, 44–47]. As the phenotype of procollagen 11A1^+^ “myofibroblasts” from colon adenocarcinoma resembles that of cultured TGF-β1-activated human bone marrow mesenchymal cells, all these observations add support to the tenet that at least some cancer-associated stromal cells, such as the procollagen 11A1^+^ ones, could be bone marrow-derived mesenchymal cells. Altogether, we may suggest that procollagen 11A1 could be expressed by a more specialized subpopulation among “activated myofibroblasts”.

Halsted *et al.* [14] reported the cytoplasmic, stromal and vascular immunostaining of both normal and malignant human breast tissues with polyclonal antisera to specific regions of N-terminal domains of human procollagen 11A1. Vargas *et al*. [30] immunodetected collagen 11A1 in the normal epithelium of human breast and Wu *et al.* [31] performed it in some human ovarian cancer cell lines, after applying another antibody preparation. Moreover, rather contradictory observations have been reported in relation to the kind of cells in which *COL11A1* mRNA has been detected. While *in situ* hybridization studies have spotted its detection only in stromal cells [1, 17], another study, based on differentially expressed gene analyses by *GeneChip* hybridization, has pointed to the over-expression of *COL11A1* mRNA in tumour epithelia [13]; very recently, Cheon *et al.* [48] have reported, also through *in situ* hybridization and immunohistochemistry with the DMTX1/1E8.33 mAb of serous ovarian cancer, that “COL11A1 expression was confined to intra/peritumoral stromal cells and rare foci of tumor epithelial cells”.

In our experience, the immunodetection of procollagen 11A1 with the DMTX1/1E8.33 mAb has never been observed in normal epithelial, vascular or stromal cells but in cancer-associated stromal cells; immunochemistry discrepancies between our observations and those above mentioned may be attributed to the different fine specificity of the applied antibody preparations. Besides this, transcription profiling studies of human colon biopsies obtained from active and inactive areas of ulcerative colitis and Crohn’s disease, compared with samples from infectious colitis and healthy controls, have shown that there are no differences in the expression levels of the *COL11A1* gene between any of the above referred to conditions [49–53]. *COL11A1/*procollagen 11A1 expression is mostly absent in benign inflammatory processes such as breast sclerosing adenosis [16, 54], chronic pancreatitis [41], and diverticulitis (our own observations; data not shown), and is rather low in familial adenomatosis polyposis adenomas [1, 2]. Thus, the *in vivo* up-regulation of the *COL11A1* gene may be considered as a biomarker of cancer-associated stromal cells.

In this study, high procollagen 11A1 immunostaining was associated with clinicopathological variables such as lymph node involvement, advanced Dukes stages and presence of distant metastases. These results go according to the role of *COL11A1* in promoting carcinoma aggressiveness and progression [11, 17, 20, 30, 31, 48, 55–57].

## Conclusions

Based on its high specificity, our observations stress once more the usefulness of the DMTX1/1E8.33 mAb for cancer research, and the clinical significance of procollagen 11A1 as a very valuable biomarker to characterise cancer-associated stromal cells and to evaluate human colon adenocarcinomas.

## Electronic supplementary material

Additional file 1: **Detailed description of patients and their clinicopathological characteristics.** (PDF 25 KB)

## Abbreviations

HMCs: Human mesenchymal cells
hTERT-HMCs: Immortalised hTERT- human bone marrow mesenchymal cells
ICC: Immunocytochemistry
IHC: Immunohistochemistry
mAb: Monoclonal antibody
pAb: Polyclonal antibodies
H&E: Hematoxylin and eosin
PBS: Phosphate-buffered saline
DAB: 3-3′-Diaminobenzidine.
