# Supplementary material for: Validation of COL11A1/procollagen 11A1 expression in TGF-β1-activated immortalised human mesenchymal cells and in stromal cells of human colon adenocarcinoma
Source: BMC Cancer. 2014 Nov 23;14:867. doi: 10.1186/1471-2407-14-867 (PMC4246482; doi:10.1186/1471-2407-14-867)
Supplement: Supplementary file 1 — Additional file 1: Detailed description of patients and their clinicopathological characteristics. (PDF 25 KB) [file 12885_2014_5037_MOESM1_ESM.pdf]

Patient	Tumor size (cm)	Tumor localization	Tumor differentiation	T	N	M	Extent of immunostaining	Granularity of immunostaining	Immunoscore
1	4.9	S	MD	T3	N0	M0	Negative	Negative	0
2	6.0	AC	PD	T3	N1	M1	>50%	Confluent	6
3	9.0	DC	PD	T4	N1	M1	Negative	Negative	0
4	11.0	AC	MD	T4	N0	M0	Negative	Negative	0
5	2.0	S	MD	T3	N0	M0	<10%	Dispersed	1
6	3.0	AC	WD	T1	N0	M0	<10%	Dispersed	1
7	2.5	S	MD	T2	N0	M0	<10%	Dispersed	1
8	1.5	S	WD	T1	N0	M0	<10%	Confluent	2
9	0.5	DC	WD	T2	N0	M0	10-50%	Dispersed	2
10	4.0	AC	WD	T2	N0	M0	10-50%	Dispersed	2
11	4.5	S	MD	T1	N0	M0	10-50%	Dispersed	2
12	3.7	AC	WD	T4	N0	M0	>50%	Confluent	6
13	3.0	S	WD	T3	N0	M0	>50%	Dispersed	3
14	4.0	S	MD	T3	N0	M0	<10%	Dispersed	1
15	2.0	AC	WD	T3	N0	M0	<10%	Dispersed	1
16	2.8	AC	WD	T2	N0	M0	>50%	Confluent	6
17	3.0	S	MD	T4	N1	M0	>50%	Confluent	6
18	3.5	DC	PD	T4	N1	M0	>50%	Confluent	6
19	4.0	DC	WD	T3	N1	M0	10-50%	Dispersed	2
20	5.0	AC	WD	T3	N1	M0	>50%	Dispersed	3
21	4.0	DC	WD	T2	N1	M0	10-50%	Dispersed	2
22	1.0	S	WD	T2	N0	M0	<10%	Dispersed	1
23	1.5	S	WD	T3	N0	M0	10-50%	Dispersed	2
24	3.5	S	WD	T3	N0	M0	>50%	Confluent	6
25	4.0	AC	MD	T3	N1	M0	10-50%	Dispersed	2
26	1.0	DC	MD	T4	N1	M0	10-50%	Confluent	4
27	3.5	DC	MD	T4	N1	M0	<10%	Dispersed	1
28	4.0	S	MD	T4	N1	M0	10-50%	Dispersed	2
29	2.0	S	MD	T3	N1	M0	<10%	Dispersed	1
30	2.5	AC	WD	T3	N1	M0	<10%	Dispersed	1
31	5.5	S	WD	T3	N1	M1	>50%	Confluent	6
32	4.5	S	MD	T3	N1	M1	10-50%	Confluent	4
33	3.5	S	MD	T4	N1	M1	<10%	Dispersed	1
34	5.0	AC	WD	T4	N0	M0	10-50%	Dispersed	2
35	3.0	S	MD	T4	N1	M1	>50%	Dispersed	3
36	4.5	S	WD	T3	N0	M0	<10%	Dispersed	1
37	4.5	AC	MD	T3	N1	M0	10-50%	Dispersed	2
38	6.0	AC	MD	T3	N0	M0	>50%	Confluent	6
39	5.5	DC	MD	T3	N0	M0	<10%	Dispersed	1
40	2.5	S	WD	T2	N0	M0	10-50%	Confluent	4
41	0.9	S	MD	T3	N1	M1	>50%	Confluent	6
42	4.0	AC	MD	T3	N0	M1	10-50%	Dispersed	2
43	8.0	S	MD	T3	N1	M1	>50%	Confluent	6
44	4.0	AC	PD	T3	N1	M1	>50%	Dispersed	3
45	3.5	AC	MD	T3	N0	M0	>50%	Confluent	6
46	2.5	AC	MD	T3	N1	M1	10-50%	Confluent	4
47	4.0	S	MD	T3	N1	M0	10-50%	Dispersed	2
48	6.5	AC	MD	T4	N1	M1	>50%	Confluent	6
49	3.0	AC	MD	T3	N1	M0	10-50%	Confluent	4
50	8.5	AC	MD	T4	N0	M0	10-50%	Confluent	4

51	3.0	AC	MD	T3	N1	M0	>50%	Confluent	6
52	Ischemia						Negative	Negative	0
53	Ischemia						Negative	Negative	0
54	Ischemia						Negative	Negative	0
55	Ischemia						Negative	Negative	0
56	Ischemia						Negative	Negative	0
57	Ischemia						Negative	Negative	0

AC: Ascending colon; DC: Descending colon; S: Sigmoid; WD: Well differentiated; MD: Moderately differentiated: PD: Poorly differentiated
